# Primary Cilia Are Not Required for Normal Canonical Wnt Signaling in the Mouse Embryo

**DOI:** 10.1371/journal.pone.0006839

**Published:** 2009-08-31

**Authors:** Polloneal Jymmiel R. Ocbina, Miquel Tuson, Kathryn V. Anderson

**Affiliations:** 1 Developmental Biology Program, Sloan-Kettering Institute, New York, New York, United States of America; 2 Neuroscience Program, Weill Cornell Graduate School of Medical Sciences, Cornell University, New York, New York, United States of America; Max Planck Institute of Molecular Cell Biology and Genetics, Germany

## Abstract

**Background:**

Sonic hedgehog (Shh) signaling in the mouse requires the microtubule-based organelle, the primary cilium. The primary cilium is assembled and maintained through the process of intraflagellar transport (IFT) and the response to Shh is blocked in mouse mutants that lack proteins required for IFT. Although the phenotypes of mouse IFT mutants do not overlap with phenotypes of known Wnt pathway mutants, recent studies report data suggesting that the primary cilium modulates responses to Wnt signals.

**Methodology/Principal Findings:**

We therefore carried out a systematic analysis of canonical Wnt signaling in mutant embryos and cells that lack primary cilia because of loss of the anterograde IFT kinesin-II motor (*Kif3a)* or IFT complex B proteins (*Ift172* or *Ift88)*. We also analyzed mutant embryos with abnormal primary cilia due to defects in retrograde IFT (*Dync2h1)*. The mouse IFT mutants express the canonical Wnt target *Axin2* and activate a transgenic canonical Wnt reporter, BAT-gal, in the normal spatial pattern and to the same quantitative level as wild type littermates. Similarly, mouse embryonic fibroblasts (MEFs) derived from IFT mutants respond normally to added Wnt3a. The switch from canonical to non-canonical Wnt also appears normal in IFT mutant MEFs, as both wild-type and mutant cells do not activate the canonical Wnt reporter in the presence of both Wnt3a and Wnt5a.

**Conclusions:**

We conclude that loss of primary cilia or defects in retrograde IFT do not affect the response of the midgestation embryo or embryo-derived fibroblasts to Wnt ligands.

## Introduction

Recent experiments have provided strong evidence that primary cilia are essential for mammalian Hedgehog signaling. The primary cilium is a highly conserved microtubule-based organelle that grows from a basal body, a modified centrosome, and projects from the cell surface into the extracellular environment and plays diverse roles in cellular motility, sensory transduction and signaling. The process of intraflagellar transport (IFT), which is necessary for the assembly and maintenance of primary cilia, is the bidirectional movement of cargo by IFT protein complexes along axonemal microtubules [reviewed in 1]. Mutations that block the formation of cilia, either by blocking IFT or through disruption of specific basal body proteins, prevent the normal regulation of Gli transcription factors in response to Hedgehog ligands [reviewed in 2], [3], [4]. Strong support for the cilia hypothesis has been provided by experiments that show that all of the core Hh signal transduction components that have been analyzed are enriched in cilia [5]–[8].

The demonstration that mammalian, but not *Drosophila*, Hedgehog signaling depends on cilia has led to considerable interest in the roles cilia might play in other vertebrate signal transduction pathways. In particular, a number of studies have suggested that there is a connection between cilia and Wnt signaling [9]–[11]. The first observation that suggested a connection between primary cilia and Wnt signaling came from the demonstration that Inversin regulates levels of Disheveled (Dvl) and can act as a switch between canonical and non-canonical Wnt pathways [11]. Inversin protein is enriched in cilia and basal bodies (in addition to the adherens junctions and nucleus [12]), which suggested that cilia might provide an important site for localization of Wnt pathway components.

Two recent studies reported that disruption of ciliogenesis causes hyper-responsiveness to canonical Wnt signals in vertebrates [9], [13]. These findings have elicited considerable interest in the community [14], [15]. However, the phenotypes of mouse mutant embryos that lack cilia do not overlap with the phenotypes of Wnt pathway mutants; and mutants that lack cilia because of a mutation in the IFT B complex protein IFT172 have normal canonical Wnt signaling, as assayed by a Wnt reporter transgene [2], [16] Mutant mouse embryos that lack IFT and primary cilia die between 9 and 11 days of gestation with characteristic defects in the morphology of the developing nervous system. It is therefore possible that the early lethality and/or morphological abnormalities of IFT mutant embryos might mask a subtle role of cilia in the Wnt pathway. We have therefore carried out a systematic analysis of canonical Wnt signaling in embryos and cells that are mutant for four different IFT proteins, using four different molecular assays.

## Results

### *Axin2* is expressed normally in IFT mutant embryos

**Figure 1:**
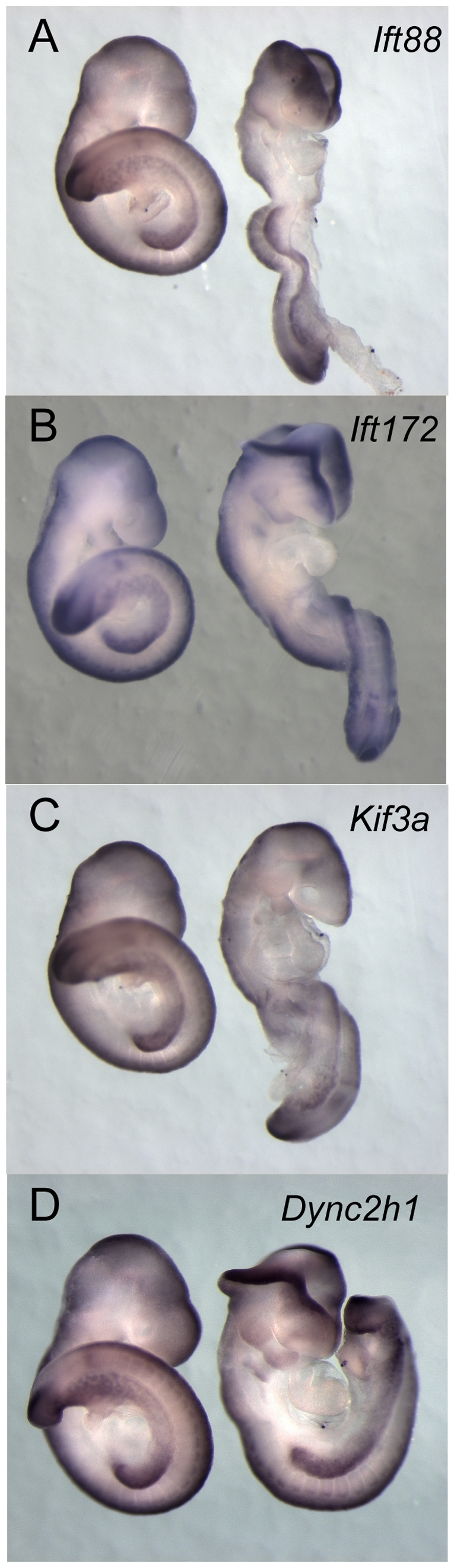
*Axin2* transcripts are expressed normally in IFT mutants. Wholemount *in situ* hybridization for *Axin*2, a downstream target of canonical Wnt signaling, in wild type and IFT mutant embryos. The domains of expression of *Axin* in *Ift88* (A), *Ift172* (B), *Kif3a* (C) and *Dync2h1* (D) mutant embryos were indistinguishable from those of wild-type littermates.

To test for a role of primary cilia in Wnt signaling during development, we analyzed the expression of *Axin2* in wild-type and mutant midgestation (embryonic day 9.5 (e9.5)) mouse embryos. The *Axin2* gene is a direct transcriptional target of the canonical Wnt pathway that is expressed in embryonic cells where canonical Wnt signaling is active [17], [18]. At e9.5 *Axin2* transcripts were detected in the otic vesicle, dorsal neural tube, the branchial arches, somites, limb buds and tail bud of wild type embryos as previously described (Figure 1) [17], [18].

Because complex mechanisms control ciliogenesis, we analyzed the requirement of cilia for *Axin2* expression in mutants in four genes that affect different aspects of IFT. *Kif3a* encodes a subunit of the kinesin-II motor that drives anterograde trafficking from the base to the tip of the cilium and *Kif3a* mutants do not generate cilia [3], [19], [20]. Similarly, mouse mutants that lack IFT complex B proteins IFT172 or IFT88 do not make cilia [3], [21], [22]. *Dync2h1* encodes the heavy chain of the cytoplasmic dynein that is required for retrograde intraflagellar transport [23], [24]. By whole mount *in situ* hybridization, we found that both wild-type and all four IFT mutants expressed *Axin2* prominently in the dorsal neural tube, tail bud, limb buds and somites (Figure 1). No difference was detected in either the position or level of expression of *Axin2* expression among the genotypes, although the IFT mutants showed the defects in the morphology of the neural tube and embryonic turning that have been attributed to defects in Shh signaling (Figure 1A–D). Thus by this assay, mutations in four different genes that affect different aspects of IFT have no detectable effect on canonical Wnt signaling.

### A canonical Wnt reporter is expressed in the normal pattern in IFT mutant embryos

**Figure 2:**
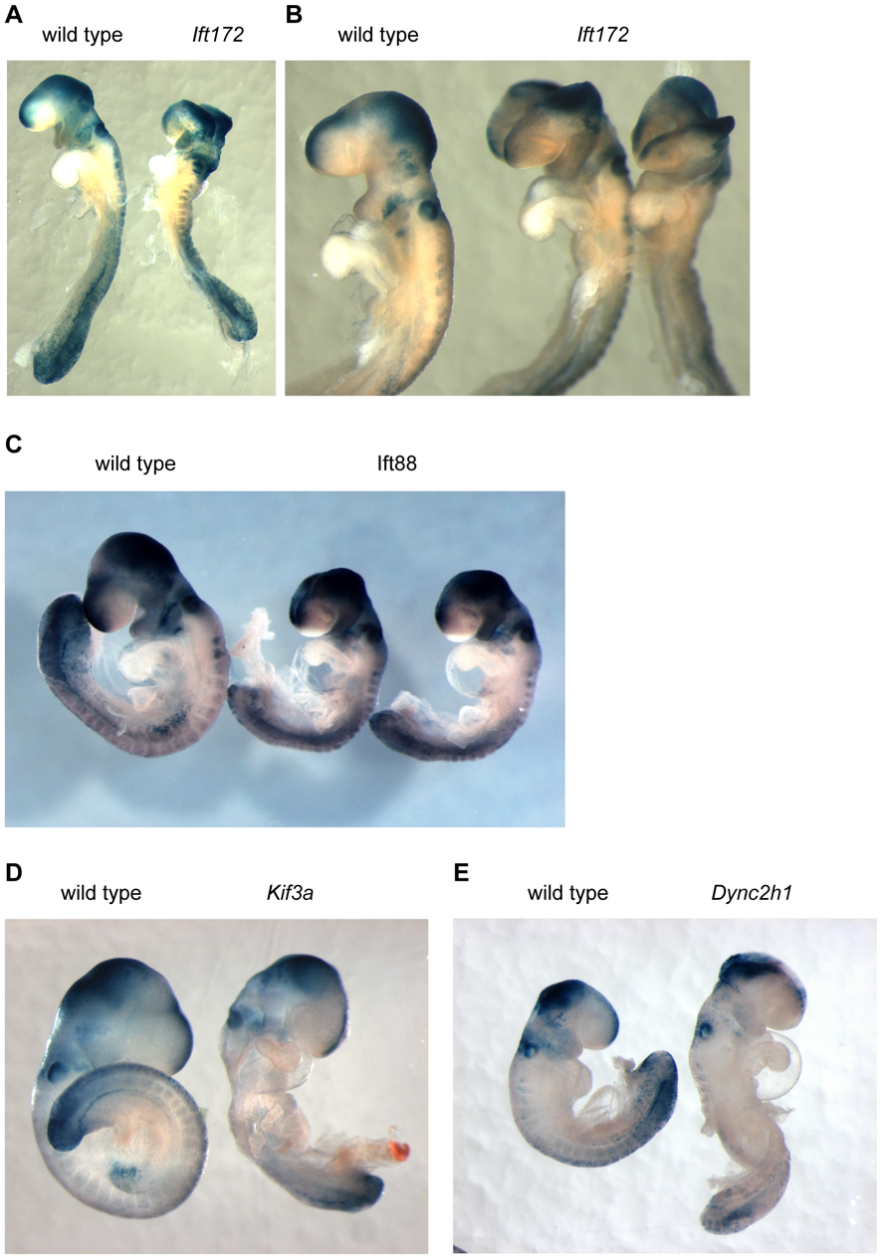
Normal spatial pattern of canonical Wnt response in IFT mutants. The BAT-gal transgene expresses β-galactosidase under the control of seven tandem TCF/LEF sites that mediate responses to canonical Wnt signals. A. Reporter expression patterns are similar in e9.0 wild type and *Ift172* mutant embryos that carry one copy of the BAT-gal reporter. B. Higher magnification view of the anterior of wild type and mutant embryos shows indistinguishable patterns and levels of reporter expression. BAT-gal reporter expression in *Ift88* (C), *Kif3a* (D) and *Dync2h1* (E) mutant embryos with wild type littermates at e9.5.

We previously showed that a canonical Wnt reporter, TOP-gal, was expressed in the normal pattern in *Ift172* embryos at e10.5 [2]. To assess the role of primary cilia in regulating canonical Wnt signaling during early mouse development more stringently, we analyzed earlier embryos and used a more sensitive reporter of canonical Wnt activity, the BAT-gal transgene [25], which contains seven tandem Lef/Tcf binding sites that drive the expression of β-galactosidase in response to canonical Wnt signals. At e9.5, *Ift172* mutants carrying the BAT-gal transgene showed the abnormal neural tube morphology characteristic of mutants that lack Sonic hedgehog (Shh)-dependent ventral neural cell types. Despite their abnormal cranial morphology, the spatial pattern of BAT-gal reporter expression was normal in *Ift172* embryos and was indistinguishable from that of wild type littermates (Figure 2A,B).

Embryos homozygous for a targeted null allele of *Ift88* lack all cilia, like *Ift172* mutants [26]. While IFT172 appears to be a peripheral component of IFT complex B [27], IFT88 is a core IFT complex B component. Homozygous *Ift88*-null mutant e9.5 embryos carrying one copy of the BAT-gal reporter expressed β-galactosidase in a pattern indistinguishable from that of wild type littermates (Figure 2C). *Kif3a* mutants, which also lack all cilia due to the absence of the Kinesin-II anterograde IFT motor, arrest at e9.0, somewhat earlier than *Ift88* and *Ift172* null mutants. *Kif3a* homozygous mutant embryos expressed the BAT-gal reporter in the same regions and at the same level as their wild type littermates (Figure 2D). In *Dync2h1* mouse mutants, which make short, bulged cilia due to a lack of retrograde IFT, the BAT-gal reporter was again expressed in a similar pattern and with the same intensity that paralleled wild type littermates (Figure 2E). Thus, using the expression pattern of this β-galactosidase reporter to assay the qualitative responses to canonical Wnt signals in the absence of cilia, we did not detect any difference in the pattern of reporter expression between wild type and mutant embryos that lack cilia altogether or have disruptions in cilia structure.

### Loss of cilia does not influence the amount of activity of a canonical Wnt reporter

**Figure 3:**
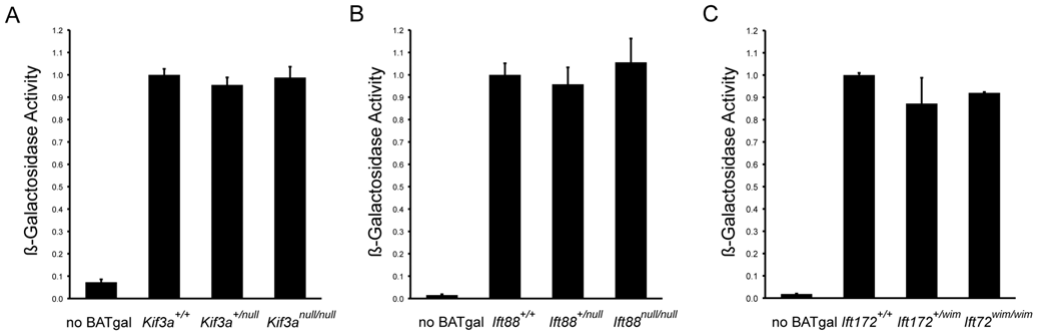
Quantitation of BAT-gal reporter in whole embryo lysates. Transcriptional activation in response to canonical Wnt signals was quantitated in e9.5 embryos that carried one copy of the BAT-gal reporter using a spectrophotometric assay for β-galactosidase enzyme activity (data are mean, ±s.d., n≥5; ANOVA, Tukey's post-hoc tests, n.s.).

In addition to providing spatial information on active canonical Wnt signaling, β-galactosidase enzymatic activity in embryos carrying the BAT-gal transgene can be used to detect subtle quantitative differences in Wnt activity. We made whole embryo extracts from e9.5 embryos that carried the BAT-gal transgene and assayed enzyme activity *in vitro* (Figure 3). There was no difference in β-galactosidase activity in extracts of e9.5 wild-type, *Kif3a*/+ and *Kif3a*/*Kif3a* homozygous mutant embryos that carried a single copy of the BAT-gal transgene (Figure 3A). Similarly, β-galactosidase activity was indistinguishable in wild-type, heterozygous and homozygous *Ift88-null* (Figure 3B), and heterozygous or homozygous *Ift172* (Figure 3C) mutant embryos carrying the BAT-gal reporter. Thus this transgenic reporter of canonical Wnt signaling demonstrates that genetic ablation of ciliogenesis does not detectably alter responses to canonical Wnt ligands in the midgestation mouse embryo.

### The response of fibroblasts to Wnt3a does not depend on cilia

**Figure 4:**
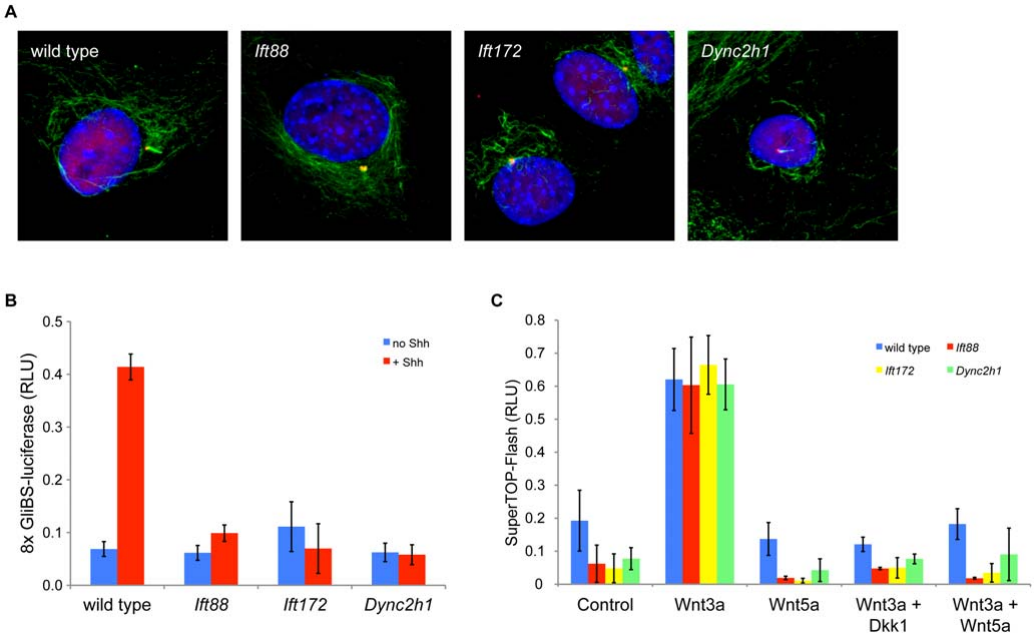
The response of fibroblasts to Wnt3a does not depend on cilia. A. Wild type, *Ift88*, *Ift172* and *Dync2h1* MEFs stained for cilia (acetylated α-tubulin, green) and basal bodies (centrin, red). MEFs derived from e9.5 wild type and *Dync2h1* embryos generate cilia within 24 hours of culture, but not *Ift172* or *Ift88* mutant MEFs. B. *Ift88*, *Ift172* and *Dync2h1* MEFs fail to respond to Shh. Cells were transfected with a Hh-responsive Gli-luciferase reporter and stimulated with Shh-enriched media. Wild-type cells showed robust activation of the reporter in response to Shh treatment, whereas *Ift88*, *Ift172* and *Dync2h1* MEFs were completely non-responsive to Shh. C. *Ift88*, *Ift172* and *Dync2h1* MEFs respond normally to Wnt3a. SuperTOP-Flash reporter activity was assayed in response to 100 ng/mL recombinant Wnt3a. Reporter activity presented as relative light units (RLU) normalized to Renilla luciferase control. *Ift172* and *Dync2h1* mutant MEFs activated the reporter in response to different levels of Wnt3a similar to wild type levels (data are mean ±s.d., n = 4).

Whole embryos are a mixture of cell types, which might obscure an abnormal response to Wnt in specific responsive tissues. To assay the response to canonical Wnt ligands in a more uniform population of cells, we generated primary mouse embryonic fibroblasts (MEFs) derived from wild-type and mutant embryos and assayed their response to a canonical Wnt ligand. MEFs derived from *Ift88* or *Ift172* embryos do not make cilia (Figure 4A) [28]. We measured the response of the mutant MEFs to Shh in *Ift88 and Ift172* mutant cells with a Gli-luciferase reporter [29]. When treated with Shh, mutant cells were completely unresponsive and failed to activate the Gli-luciferase reporter above basal levels; these findings parallel the loss of Hedgehog-responsiveness in mutant embryos (Figure 4B) [3], [26]. To measure the Wnt-responsiveness of the cells, we transfected the MEFs with the SuperTOPFlash reporter [30] and stimulated the cells with 100 ng/mL of Wnt3a (Figure 4C). Both *Ift88* and *Ift172* mutant cells activated the SuperTOPFlash reporter in response to Wnt3a to levels indistinguishable from those of wild type primary MEFs. Dkk1 is a potent inhibitor of β-catenin dependent Wnt signaling [31]. Treatment of cells with Wnt3a in the presence of 50 ng/mL of Dkk1 blocked reporter activation by Wnt3a in wild-type, *Ift88* and *Ift172* mutant MEFs (Figure 4C). Thus transcriptional activity in response to canonical Wnt ligands is normal in cells that do not have cilia. Furthermore, loss of cilia does not affect the cell's ability to respond to inhibitors of canonical Wnt signaling.

*Dync2h1* MEFs have cilia that are of approximately normal length, although they have a characteristic bulge along the axoneme due to the disruption of retrograde IFT (Figure 4A) [28]. We previously showed that *Dync2h1* MEFs fail to respond to Shh [28]. However, like the responses seen in *Ift88* and *Ift172* MEFs, *Dync2h1* MEFs activated the SuperTOPFlash reporter normally in response to recombinant Wnt3a and this response was attenuated in the presence of the canonical Wnt inhibitor, Dkk1 (Figure 4C).

### Loss of cilia does not disrupt the shift between canonical and non-canonical Wnt signaling in MEFs

All of the assays described thus far indicate that canonical Wnt signaling is normal in IFT mutants. Several observations have suggested that loss of cilia causes a loss of non-canonical Wnt signaling; in particular, it has been suggested IFT and/or basal body proteins may regulate a switch between canonical and non-canonical Wnt signaling [9]–[11]. IFT mutant embryos do not show the craniorachischisis (open neural tube caudal to the forebrain) characteristic of mouse mutants that lack components of the non-canonical Wnt pathway [32], [33], suggesting there is not a strong disruption of non-canonical Wnt signaling in IFT mutants. Although there is no single biochemical assay for the activity of the non-canonical Wnt pathway, the non-canonical Wnt ligand Wnt5a can repress β-catenin/TCF/LEF1-dependent transcription, although the mechanism remains unclear [34], [35]. We therefore tested the responses of IFT mutant MEFs to Wnt5a. As expected, stimulation with the non-canonical ligand Wnt5a did not activate the SuperTOPFlash reporter in wild-type or mutant cells (Figure 4C). When cells were stimulated with Wnt5a and Wnt3a simultaneously, canonical Wnt reporter activation was attenuated in wild-type MEFs, as previously described [35]. Wnt5a also blocked the Wnt3a-dependent induction of the SuperTOPflash reporter *Ift88*, *Ift172* and *Dync2h1* mutant MEFs, exactly as seen in wild-type cells (Figure 4C). These results demonstrate that either disruption of ciliogenesis or loss of retrograde IFT does not affect the ability of MEFs to properly modulate Wnt responses.

## Discussion

In this study, we provide genetic and biochemical evidence that canonical Wnt signaling is normal in mouse embryos and in MEFs in the absence of cilia. In contrast to earlier reports, which indicated that loss of cilia increased the response of cells to Wnt ligands, we demonstrate that mice that lack components of the IFT B complex (IFT88 and IFT172), or subunits of the motors that drive anterograde (Kif3a) or retrograde (Dync2h1) trafficking within cilia express canonical Wnt gene targets such as *Axin2* or activate the BAT-gal reporter in the normal spatial pattern. Measurement of BAT-gal activity in the embryo or the SuperTOPFlash reporter in MEFs derived from IFT mutants show that the level of response to canonical Wnt signals is not affected in the absence of IFT172, IFT88, Kif3a or Dync2h1. Based on these findings, we conclude that lack of cilia or disruption in retrograde IFT does not alter canonical/β-catenin-dependent Wnt signaling. These findings contrast with previous results that suggested that IFT proteins and, in particular, Kif3a, have specific roles in regulation of the canonical Wnt pathway [9], [13].

### IFT mutants do not show phenotypes associated with the Wnt pathway

It has previously been reported that loss of cilia leads to enhanced responses to canonical Wnt ligands. Increased canonical Wnt activity in mutants that lack negative regulators of Wnt signaling, such as Axin, APC, Dkk1 or Cer1, causes arrest during early development [36]–[38] with characteristic phenotypes that do not overlap with those of IFT mutants. *APC* mutants cause a strong activation of canonical Wnt signaling that leads to arrest prior to gastrulation [36]. A partial loss-of-function allele of APC does not cause as strong an activation of canonical Wnt signaling and allows survival to later stages, when embryos show axis duplications, abnormal development of the foregut and heart, and lack anterior parts of the forebrain [39], a phenotype similar to mutants that lack the activity of Axin, another negative regulator of the Wnt pathway [37], [40]. Mutants that lack the canonical Wnt antagonist Dkk1 show a weaker phenotype, but still lack anterior parts of the brain [38]. In contrast, IFT mutants survive to e10.5, never show axis duplications, show normal patterning of the gut and heart and specify the forebrain normally. *Kif3a* mutants arrest at e9.0, earlier than other IFT mutants, probably due to roles of Kinesin-II outside of cilia [41], [42]. *Kif3a* mutants lack the Shh-dependent ventral neural types, but do not have axis duplications, defects in ventral morphogenesis or anterior neural truncations (Figure 1 and 2). Thus the phenotypes of IFT null mutant embryos do not reveal any elevation of canonical Wnt signaling in the absence of cilia or when cilia structure is disrupted. Thus both the phenotype of IFT null mutant embryos and the reporter assays presented here show that canonical Wnt signaling is normal in the absence of cilia.

### Cilia and the PCP pathway

The data that cilia are positive regulators of non-canonical Wnt signaling are more complex. The non-canonical arm of the Wnt pathway regulates the planar cell polarity (PCP) pathway in the mouse [reviewed in 43]. Observations in cultured cells, as well as in mouse, zebrafish and frog embryos, have implicated IFT and basal body proteins in regulating the switch between canonical and non-canonical Wnt signaling [9]–[11]. However, by one biochemical assay, the ability of a non-canonical ligand to block responsiveness to a canonical Wnt, we find that the switch between canonical and non-canonical pathways is normal in IFT mutant fibroblasts.

Mouse mutant embryos that lack components of IFT or components of the non-canonical Wnt pathways have non-overlapping phenotypes. Mouse embryos that have mutations in core non-canonical Wnt genes such as *Vangl2* and *Celsr1* have a shortened body axis and fail to initiate neural tube closure in the hindbrain and spinal region, although the neural tube in the forebrain is closed. At the end of gestation, these mutants have a severe malformation of the neural tube similar to severe craniorachischisis defects in humans [32], [33]. Even though the neural tube is completely open in *Vangl2/Lp* mutants, patterning of the neural tube is fairly normal, with slight expansion of the floor plate and mild dorsal expansion of the expression domain of *Pax6* [33]. In contrast, IFT mutants do not exhibit craniorachischisis (Figure 1 and 2). Instead, depending on the genetic background, they show exencephaly, the failure to close the neural tube in the midbrain and hindbrain, which is also seen in some Shh pathway mutants [44], [45]. It is conceivable that the disruption of Shh signaling might mask a disruption in non-canonical Wnt signaling. However, double mutants that lack both *Ift172/wim* and *Vangl2/Lp* show a simple additive phenotype (K. Liem and K.V. Anderson, unpublished data), which indicates that loss of Hh signaling does not mask defects caused by a loss of non-canonical Wnt signaling.

Our findings indicate that neither canonical nor non-canonical Wnt signaling depend on cilia in the midgestation mouse embryo. Our findings do not rule out the possibility that Wnt signaling in specialized cell types or in other animals could be modulated by ciliary components or that basal body components could have roles in Wnt signaling [9]. It has been reported that zebrafish embryos in which cilia proteins are down-regulated by treatment with morpholinos show defects in convergent extension, a process that depends on non-canonical Wnt signaling [9], [46], [47]. Mutations in two zebrafish genes that encode proteins enriched in ciliated cells affect PCP and lead to cystic kidneys, but do not disrupt ciliogenesis [48]–[50]. In the mouse inner ear, there is a complex relationship between PCP, IFT and the position of the kinocilium (a specialized primary cilium), where disruption of ciliogenesis by conditional deletion of *Ift88* reveals that this IFT protein is required to allow basal body migration in response to PCP signals [51]. Either loss of cilia [52] or loss of planar polarity [53], [54] in the kidney leads to polycystic kidney disease, suggesting that these two processes may be coupled in this tissue. We conclude that cilia are not essential for non-canonical Wnt signaling and cilia do not regulate this pathway in the mouse embryo, but additional experiments will be required to test the relationship between cilia and Wnt signaling in other animals and in the kidney.

## Materials and Methods

### Mouse Strains

Mutant alleles for *Ift88^null^*, *Ift172^wim^*, *Dync2h1^ttn^* and *Kif3a* have been previously described [3], [19], [26], [28]. Both *Kif3a-* and *Ift88*-null alleles were generated from the conditional alleles by crossing to the CAG-Cre line [55].

### *Axin2 In Situ* Hybridization

Whole mount *in situ* hybridization using an *Axin2* probe [18], [56] was performed as described previously [57].

### BAT-gal Reporter Assays

For BAT-gal experiments, males carrying one copy of the BAT-gal transgene and one copy of an *Ift88*, *Ift172*, *Kif3a* or *Dync2h1* mutant allele were crossed to females heterozygous for *Ift88*, *Ift172*, *Kif3a* or *Dync2h1* mutant allele. Embryos were dissected at e9.5 and BAT-gal activity in embryos was detected as previously described [25]. β-galactosidase activity in mouse embryos was measured using the β-galactosidase Enzyme Assay System (Promega Corporation, Madison, WI, USA) according to manufacturer's instructions. Lysates prepared from embryos were incubated at 37°C during which time β-galactosidase was allowed to hydrolyze the colorless substrate to o-nitrophenol. The reaction was stopped after 30 minutes with sodium carbonate and the absorbance was read at 420 nm with a spectrophotometer. Activity was normalized to total protein concentration, which was measured using the Pierce BCA Protein Assay Kit (Pierce Biotechnology, Rockford, IL, USA). Each column represents the mean value of at least n≥5 embryos. Data were analyzed by one-way ANOVA with Bonferoni correction and Tukey's post-hoc test. Relative β-galactosidase enzyme activities in each mutant genotype are normalized with respect to averaged wild type maximum activity levels.

### Immunostaining

Confluent MEFs were grown on gelatin-coated glass coverslips and treated with low-serum medium (0.5% bovine calf serum). After 48 h, cells were washed in PBS, fixed in 4% paraformaldehyde in PBS for 10 min on ice, permeabilized in 0.2% Triton X-100/PBS for 10 min and blocked in 0.2% Triton/2% BSA/1% FCS/PBS for 30 min at room temperature. Cells were incubated with primary antibodies against acetylated alpha tubulin (mouse 1∶1000, Sigma) and Centrin (rabbit, 1∶200, Sigma) diluted in blocking solution at 4°C overnight. Cells were washed three times in 0.02% Triton X-100/PBS and incubated with the secondary antibodies mouse Alexa 488 (1∶200) and rabbit Alexa 568 (1∶200) along with DAPI (1∶200) for 1 h at room temperature. Cells were washed as before and mounted in VectaShield (Vector Laboratories, Burlingame, CA). Confocal microscopy was performed using an upright Leica TCS SP2 AOBS laser scanning microscope. Images were taken with a 63X water objective and 1X zoom. Extended views of the confocal datasets were processed using the Volocity software package (Improvision).

### Luciferase Reporter Activity Assays

MEFs were isolated from e9.5 wild type, *Ift172^wim^*, *Ift88^null^* and *Dync2h1^ttn^* mouse embryos and cultured under standard conditions. Shh activity assays in MEFs were performed as described previously [28]. To assay for Wnt activity, MEFs were plated at a density of 1×10^5^ cells/cm^2^ in 24-well dishes the day prior to transfection. Cells were transfected 18–24 h after plating with 240 ng of the SuperTOPFlash reporter [58] and 10 ng pRL-TK (Clontech) using Fugene 6 (Roche) transfection reagent in a 3∶1 (v/w) ratio of reagent to DNA. After cells had reached confluency, they were changed to low-serum medium (0.5% bovine calf serum) for 24 h to induce cilia growth and then treated with 100 ng/mL recombinant mouse Wnt3A (R&D Systems, Minneapolis, MN, USA) alone or in combination with 50 ng/mL Dkk1 (R&D Systems, Minneapolis, MN, USA) Wnt5a [35] for 18 h. Cells were lysed after this treatment and luciferase activity was assayed with the Dual Glo Luciferase Assay (Promega). Reporter expression was normalized to cotransfected Renilla luciferase. Data were analyzed by one-way ANOVA with Bonferoni correction and Tukey's post-hoc tests.
